# The utilization of psychiatric and psychotherapeutic services in Germany – individual determinants and regional differences

**DOI:** 10.17886/RKI-GBE-2017-122.2

**Published:** 2017-12-13

**Authors:** Alexander Rommel, Julia Bretschneider, Lars Eric Kroll, Franziska Prütz, Julia Thom

**Affiliations:** Robert Koch Institute, Department of Epidemiology and Health Monitoring, Berlin

**Keywords:** MENTAL HEALTH, UTILIZATION, ACCESS TO HEALTH CARE, HEALTH SURVEY, GERMANY

## Abstract

In Germany, the provision of health services to people with mental disorders is an issue that is subject to controversial debate. On the one hand, regional differences exist in the distribution of psychotherapists in Germany. On the other hand, patients are often willing to accept the extra effort of having to travel further in order to access treatment even in case of a low supply. Thus, in addition to issues of access, an analysis of care provision also needs to take into account the actual level of services utilization. The present paper analyses the utilization of outpatient psychiatric and psychotherapeutic services and identifies individual and regional determinants.

The German Health Update (GEDA) is a nationwide survey of the adult population that is conducted by the Robert Koch Institute in the context of its population-based health monitoring. The GEDA 2014/2015-EHIS study (n=24,016) is based on a two-stage stratified random sample drawn from the population registers of 301 local authorities in Germany. The main outcome is the utilization of psychotherapeutic or psychiatric services during the last 12 months. In addition to the consideration of individual factors, the survey data was combined with information describing the regional distribution of providers of outpatient psychotherapeutic and neurological care. The data was analysed using logistic multi-level regression.

In Germany, 11.3% of women and 8.1% of men report that they have used psychotherapeutic or psychiatric treatment within the last 12 months. Among respondents with current depressive symptoms, these rates are 35.0% in women and 31.0% in men. This means that approximately two thirds of people with current depressive symptoms do not seek the services of these health professionals during this period. Apart from current depressive symptoms the utilization of psychiatric and psychotherapeutic services is associated with not living with a partner and with low levels of social support. Furthermore, in regions with a high density of care providers, the proportion of people with current depressive symptoms using such services is about 15 percentage points higher than in regions with a low density.

The conditions for the utilization of the respective services should not only be improved by increasing the number of care providers, but also by implementing accompanying measures. Innovations in health care aiming at rapid and low-threshold access as well as approaches for a better cooperation between primary and specialist care should therefore be evaluated regarding their contribution to an improved early treatment.

## 1. Introduction

**GEDA 2014/2015-EHIS**

**Data holder:** Robert Koch Institute

**Aims:** To provide reliable information about the population’s health status, health-related behaviour and health care in Germany, with the possibility of a European comparison

**Method:** Questionnaires completed on paper or online

**Population:** People aged 18 years and above with permanent residency in Germany

**Sampling:** Registry office sample; randomly selected individuals from 301 communities in Germany were invited to participate

**Participants:** 24,016 people (13,144 women; 10,872 men)

**Response rate:** 26.9%

**Study period:** November 2014 - July 2015

**Data protection:** This study was undertaken in strict accordance with the data protection regulations set out in the German Federal Data Protection Act and was approved by the German Federal Commissioner for Data Protection and Freedom of Information. Participation in the study was voluntary. The participants were fully informed about the study’s aims and content, and about data protection. All participants provided written informed consent.

More information in German is available at www.geda-studie.de

According to the Global Burden of Disease study, mental disorders are among the most frequent causes of health-related limitations throughout the world (measured in years lived with disability) and contribute significantly to the burden of disease [1, 2]. In Germany depression is the third most common cause of health-related limitations [3]. This is not only due to the prevalence of depressive disorders, but because they can considerably reduce health-related quality of life as they often appear at a young age, can recur episodically and may even develop into chronic disorders.

The spread of depression and its related consequences also pose a particular challenge to the health care system in Germany. Health insurers report a steady increase in medical diagnoses of mental disorders, in general, and depression in particular [4-8]. Depression is also associated with a noticeable increase in workplace absenteeism: although depression-related absenteeism only affects a small proportion of insured employees [8], its long duration means it is one of the conditions with the highest number of days absent from work. Furthermore, increasing numbers of people are claiming disability pension benefits due to mental disorders, with the number of claimants doubling between 1993 and 2015 [9, 10]. In the interest of public health, therefore, it is essential to understand the impact that these developments are having on the shape of the care system. This presupposes a nuanced view of the utilization of psychiatric and psychotherapeutic services.

High quality care has to ensure that people with mental disorders who have a need for assistance do indeed receive appropriate diagnosis and treatment. Before this can happen, however, the individuals in question need to enter the medical system; the first port of call, therefore, is in many cases a general practice. Importantly, according to the clinical guidelines in the long-term patients with depression should be cared for by psychotherapeutic or psychiatric specialists [11]. However, claims data shows that this is rarely the case. About 82% of patients who have been medically diagnosed with an affective disorder are cared for in general practices or practices focused on somatic disorders; among people with severe depression this still applies to 40% [12].

This situation may be partly caused by limited access to appropriate health care. The extent to which outpatient treatment in Germany reflects a patients’ needs is a contentious issue. In accordance with a strict reading of the needs-based planning directive, only a few regions can be said to be undersupplied by neurologists/psychiatrists and psychotherapists [13]. Conventional needs planning is usually used to assess the extent to which a region exceeds or falls short of the ratio of physicians per inhabitant as stipulated in the needs-based planning directive. However, since these ratios are based on historical and purely descriptive physician-inhabitant ratios, they are only partly applicable to assessments of the extent to which care provision reflects patients’ needs. Empirical studies show that the considerable regional differences in the density of outpatient specialist practices and psychotherapists do not reflect the regional differences in the frequency of mental disorders. Rather, they constitute real differences in care provision, which, in turn, lead to regional differences in access to treatment [14-19]. Moreover, difficulties in assessing the supply situation are compounded by the fact that depression can vary among patients, even among those who have received the same diagnosis [20]. The need for care can vary according to the severity of the symptoms, the limitations they place on people’s lives, the suffering a person experiences and a patient’s preferred form of treatment.

However, there are various reasons why a regionally unequal distribution of care structures need not necessarily lead to a lower demand for services or even poorer quality of care provision. Initially, this situation merely means that people face unequal access to treatment that may be perceived as a barrier to care. However, the actual utilization of health care services by people with a medical need has to be analysed separately [21]. These patients may accept different levels of effort and costs in their attempts to overcome barriers to care in order to benefit from a particular service. For example, it has been shown that among patients with pulmonary disease and multiple sclerosis, factors such as a longer distance to a physician’s practice or greater travelling time, which are the direct result of a lower supply density, have no influence on the actual utilization of necessary outpatient medical services [22]. In addition, a hypothesis has been put forward that higher demand in better-served regions does not necessarily imply better provision. Rather, it may actually be leading to supplier-induced demand; in other words, a situation in which services are used despite the lack of a need for treatment [16, 20, 23].

Therefore, when analysing the possible effects of regional disparities in care provision, alongside access, the need for medical care and levels of realised access (utilization) need to be taken into account. Importantly, utilization is not only driven by local conditions, but also by a large number of individual factors [24-26]. Some of these central socio-demographic and social determinants are considered in this study, which is aimed at describing the frequency and influencing factors that affect the demand for outpatient psychotherapeutic and psychiatric services among the general population. It is particularly focused on the question of how strongly regional differences in care affect whether a person with current depressive symptoms contacts the relevant health care professionals. The contribution closes by placing its findings within the context of current scientific and health policy discussions.

## 2. Method

### 2.1 Data

The German Health Update (GEDA) study is a nationwide survey of the adult population in Germany conducted as part of the health monitoring framework undertaken by the Robert Koch Institute (RKI) on behalf of the German Federal Ministry of Health. The questionnaire used in the European Health Interview Survey (EHIS, Wave 2) was integrated into GEDA 2014/2015 for the first time. The study collects data using a self-administered questionnaire that can be completed on paper or online. The GEDA 2014/2015-EHIS study is based on a two-stage stratified cluster sample. 301 municipalities were randomly selected for the first phase. These belong to 231 districts that represent the various municipalities and regions in Germany. People with permanent residence in one of the selected locations were randomly drawn from local population registers. A detailed description of the methodology used for GEDA 2014/2015-EHIS can be found in Lange et al. 2017 [61] as well as in the article German Health Update: New data for Germany and Europe in issue 1/2017 of the Journal of Health Monitoring and the infobox.

At the district level, the survey data were combined with information from the Federal Registry of Physicians on the regional distribution of providers of outpatient specialist care for mental disorders from the year 2013 [28]. This included data on the regional density (service providers per 100,000 inhabitants) of ‘psychotherapists’ and ‘neurologists/psychiatrists’ as defined by the needs-based planning directive [29]. Consequently, ‘psychotherapists’ are 1) predominantly or exclusively psychotherapeutic physicians 2) specialists for psychotherapeutic medicine, 3) specialists for psychosomatic medicine and psychotherapy, 4) psychological psychotherapists or 5) paediatric psychotherapists. ‘Neurologists/psychiatrists’ consist of health care providers with a specialist qualification in 1) psychiatry, 2) psychiatry and psychotherapy 3), neurology and psychiatry or 4) neurology.

In order to take into account the impact of the provision of services to neighbouring districts, supply densities were adjusted in accordance with the relations of co-provision described by the Zentralinstitut für die kassenärztliche Versorgung (Zi); these are based on the claims data covering service provision in 2008 [30, 31]. For example, service providers in core cities are frequently consulted by patients from the surrounding area (with lower levels of care provision) and thus are not fully available to the people living in core cities. As such, the ratio of physicians to the population in core cities was adjusted downwards depending on the level of service provision to the surrounding area; at the same time, the ratio of physicians to the population in the surrounding area was increased. Finally, although the distances that patients have to travel to care facilities can differ depending on their district (due to their location and the transport available), the average values in density and co-provision could not be adjusted to reflect this variation.

### 2.2 Indicators

The target variable at the individual level is the utilization of relevant specialist medical services (‘yes’/‘no’). As such, participants in the GEDA 2014/2015-EHIS study were asked: ‘Have you visited a psychologist, psychotherapist or psychiatrist in the past 12 months for consultation, examination or treatment?’ This question forms part of the catalogue of questions set out in the European Health Interview Survey (EHIS). A 2013 EU regulation made the implementation of EHIS mandatory for all European Union member states [27, 32].

In addition to age, gender, and the distinction between ‘new German federal states’ (East Germany) and ‘old German federal states (West Germany) (including Berlin)’, social indicators were selected that either reflect the social composition of the population or that previous studies have already shown to be associated with the utilization of medical services [33-36]. As with previous GEDA waves, the respondents’ socio-economic status was constructed by combining education, occupation and income into an index, and classifying the data into three status groups (‘low’, ‘medium’ and ‘high’) [37]. The respondents’ type of insurance was categorised as either ‘statutory’ or ‘private’. The indicators on partnership and social support enabled the respondents’ level of social integration to be included in the analysis. Data on partnerships was gathered using the question: ‘In your household, do you live together with someone in marriage/consensual union?’ The level of perceived social support was assessed using the Oslo-3 Social Support Scale [38]. The categories ‘medium support’ and ‘high support’ were combined, resulting in ‘low support’ and ‘medium to high support’.

Current depressive symptoms served as an indicator of a medical need for treatment. This information was gathered for GEDA 2014/2015-EHIS using self-reported data collected using the German version of the 8-item depression module that forms part of the Patient Health Questionnaire (PHQ-8) [39]. This instrument employs eight individual items that assess the frequency of current symptoms of a major depression during the last two weeks in accordance with DSM-IV (Diagnostic and Statistical Manual of Mental Disorders, 4th Edition) with the exception of suicidality. Current depressive symptoms are prevalent in respondents with a sum of at least 10 points on a scale ranging to a maximum of 24 points [39]. This measure differs from a diagnosis based on the categorisation of depression in accordance with DSM-IV, which requires the inclusion of core symptoms and exclusionary criteria. Nevertheless, PHQ is a reliable and valid screening tool for the prediction of depression and is internationally established in clinical and population-based studies [40, 41]. As an additional indicator of a medical need for care, the existence of a chronic disease was assessed using an instrument that has been standardised at the EU level as part of the Minimum European Health Module (MEHM) [42]. Respondents were asked: ‘Do you have one or more long-term chronic diseases?’ In addition to depressive symptoms, this indicator is primarily used to identify possible co-morbidities and accounts more comprehensively for a patient’s need for treatment.

### 2.3 Statistical analysis

The analyses were conducted using STATA 14.1. All calculations were carried out with a weighting factor that corrected the sample for deviations from the population structure (as of 31 December 2014) in terms of gender, age, district type and level of education. The type of district reflects the degree of urbanisation and corresponds to the regional distribution in Germany. In the descriptive analyses, the STATA procedures SVY were used for the weighting of complex samples. In order to adequately account for influencing factors at the district level (the supply density), and in addition to the individual determinants that were included in the multivariate analysis, multi-level analyses (random-intercept regression models) were carried out using the MELOGIT procedure. Furthermore, and in line with the XTMRHO procedure, median odds ratios were calculated as a measure of the mean variation in utilization between districts [43]. Associations between determinants and utilization are reported as odds ratios. The MARGINS and MARGINSPLOT procedures were employed for model based predictions and visualisations of the utilization of psychotherapeutic and psychiatric services depending on the density of supply. For this, possible interactions between the variables were additionally considered in the model. Weighting for the multilevel models was done by decomposing the sample weights into separate weights for districts and individuals [44].

## 3. Results

### 3.1 Supply structures

**Figure 1:**
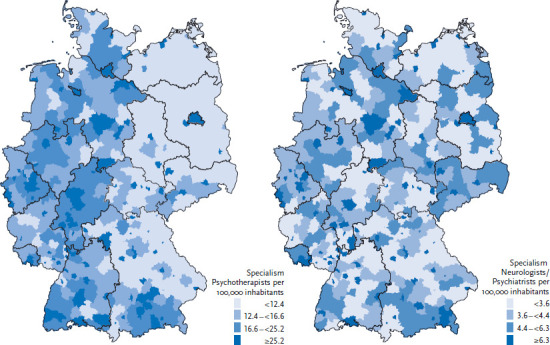
The regional distribution of psychotherapists and neurologist/psychiatrist practices in 2013 (defined in accordance with the directive for needs-based planning; raw provider density; Physicians per 100,000 inhabitants) Source: Federal Registry of Physicians [28]

An analysis of the extent to which the utilization of health services is influenced by the regional supply presupposes the existence of an unequal distribution of services. This is certainly the case in Germany. Particularly in psychotherapeutic care, a considerable regional variation in the density of outpatient care providers exists (with the group of physicians defined in accordance with the needs-based planning directive; see Method). In 2013, coverage ranged from a nominal 1.7 service providers per 100,000 inhabitants in the district of Landshut to 129.7 service providers per 100,000 inhabitants in the urban district of Heidelberg [28]. Two patterns are clear from the existing regional distribution: on the one hand, coverage is significantly lower in certain areas, especially in districts in eastern Germany and Bavaria, than in large parts of western Germany. On the other hand, there is particularly high coverage in urban districts and city-states (Figure 1). In eastern Germany, a large number of districts do not benefit from co-provision by small, medium or large cities. Since the implementation of district reforms, there are no longer any independent urban districts in the northeast of Germany; as such, the low supply densities in these regions effectively reflect the average results for urban and rural areas.

The case is different for physicians specialised in neurology/psychiatry (defined in accordance with the directive for needs-based planning; see Method). On the one hand, regional patterns, such as the difference between eastern and western Germany, are less pronounced (Figure 1). On the other hand, the supply density is significantly lower than for psychotherapists. In fact, in some districts, such as Schweinfurt there are no specialist neurological/psychiatric practices at all. At the same time, the differences in coverage are also significantly lower: the highest density of psychotherapists is found in the urban district of Bamberg with 17.9 service providers per 100,000 inhabitants.

**Figure 2:**
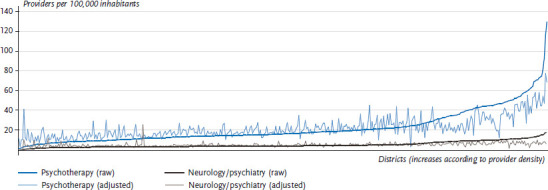
The distribution of practices providing psychotherapy and neurology at the district level in 2013 (defined in accordance with the needs-based planning directive; crude provider density, and density adjusted due to the co-provision of care) Source: Federal Registry of Physicians [28], Zi [30, 31]

The frequent proximity of less well-served rural districts to urban districts with relatively good levels of service provision highlights the need to adequately account for cross-boundary service provision (co-provision). Doing so leads to a significant reduction in regional disparities in supply. The example of psychotherapists, in particular, demonstrates that the supply in districts with a very low supply density is usually better than assumed once the co-provision of services by other districts is taken into account. In districts and cities with a comparatively good level of service provision, the co-provision of outlying regions results in a lower level of supply in these areas. This reduces the maximum number of service providers that are actually available from close to 130 per 100,000 inhabitants to around 75 (Figure 2).

### 3.2 Utilization of psychotherapeutic and psychiatric services

**Figure 3:**
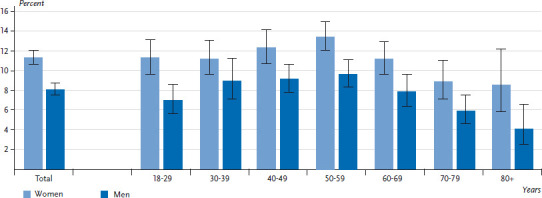
Utilization of psychotherapeutic or psychiatric services according to age and gender (n=23,875) Source: GEDA 2014/2015-EHIS

In addition to the service supply structures, an analysis of the health care situation also needs to take into account the actual utilization of the services concerned. In Germany, 9.7% (95% CI: 9.2-10.3) of the adult population report having contact with psychotherapeutic or psychiatric care providers at least once within the last year. At 11.3% (95% CI: 10.6-12.1), women are significantly more likely to visit these specialists than men, for whom the figure stands at 8.1% (95% CI: 7.5-8.8). In addition, changes also occur in the utilization of these services with increasing age (Figure 3).

The proportion of men and women who seek treatment from psychotherapeutic or psychiatric care providers initially increases with age, reaching the highest rates among adults aged between 50 and 59 (13.4% for women and 9.7% for men). After this, self-reported utilization declines, reaching the lowest figures among both genders for 70- to 79-year-olds (women: 8.9%, men: 5.9%) and respondents aged 80 or above (women: 8.5%, men: 4.1%).

A significantly higher utilization was also identified among people suffering from a chronic disease (Table 1). However, marked differences are particularly evident between individuals who present current depressive symptoms compared to people without such symptoms. 35% of women and 31% of men with current depressive symptoms report having visited a psychotherapeutic or psychiatric care provider in the past 12 months. In other words, around two thirds of people presenting current depressive symptoms did not seek psychotherapeutic or psychiatric care in the past 12 months or were treated in general practices or by care providers focused on somatic care.

Moreover, the utilization of psychotherapeutic and psychiatric care as well differs by social and socio-demographic factors (Table 1). For example, people with a low socio-economic status are more likely to visit these specialists than people with a medium or high socio-economic status. The differences identified between people with statutory or private health insurance as well as between the old and new German federal states, however, are small. In view of the lower density of care in the new federal states, at the descriptive level, this finding indicates that a lower level of supply does not directly coincide with a lower utilization (Table 1, Figure 1).

On the other hand, stronger associations exist between social inclusion and the utilization of psychotherapeutic or psychiatric services. People not living in marriage/consensual union, and those with low levels of social support in particular, are more likely than others to seek psychotherapeutic or psychiatric treatment.

### 3.3 Individual and regional factors that influence utilization

The multilevel analysis demonstrates significant differences between the districts in terms of the utilization of psychotherapeutic or psychiatric treatment (the median odds ratio of the model without control variables was 1.33). These differences are also associated with the regional distribution of care providers: the density of psychotherapists at the district level – adjusted for the co-provision of care – demonstrates a significant correlation with the utilization of psychotherapeutic and psychiatric treatment at the individual level. In fact, the probability that these services will be used increases, on average, by 0.7% with the addition of a further ten psychotherapists per district (odds ratio 1.007, Table 2). There is no comparable correlation between utilization and the regional distribution of neurologists/psychiatrists.

Alongside medical need, gender and age are also linked to differences in utilization. For example, women are more likely to seek psychotherapeutic or psychiatric services than men. However, the significant odds ratios gained from the analysis of age and squared age indicate that the increased utilization by age is not a linear progression (Table 2). As such, the model confirms the descriptive analysis in that utilization initially increases with age before decreasing (Figure 1). In terms of the other non-medical factors, the multivariate model mainly seems to indicate an association between social inclusion and utilization: those who do not live in marriage/consensual union, as well as those with low levels of social support, use psychotherapeutic and psychiatric treatment services significantly more often. Nevertheless, there is no significant correlation between socio-economic status or type of insurance and utilization once medical need has been controlled for.

**Figure 4:**
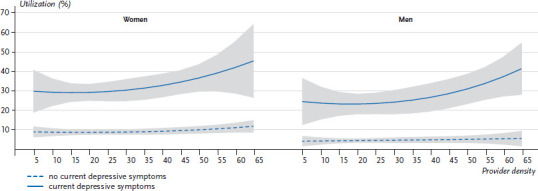
Utilization of psychotherapeutic or psychiatric services by people with and without current depressive symptoms; model based predictions with 95% confidence intervals (n=21,968) Source: GEDA 2014/2015-EHIS

Based on the models, the correlation between the regional distribution of psychotherapists and the utilization of psychotherapeutic and psychiatric services can be presented in two separate graphs for men and women. This demonstrates an evident association especially for people who had current depressive symptoms at the time of the survey (Figure 4). Among people with no current depressive symptoms, utilization stagnates with increasing supply density and remains at an almost constant, low level. Among people with current depressive symptoms, however, an increase in utilization is evident when a higher density of service provision becomes available. However, the correlation between the density of service providers and actual utilization is weaker in areas with low to medium service densities than in areas with high to very high densities. Importantly, adjusted for co-provision, only about 25% of districts have more than 35 psychotherapists per 100,000 inhabitants, and fewer than 10% have more than 50 per 100,000 inhabitants. Overall, an increased utilization of around 15 percentage points was identified. Whereas among women with current depressive symptoms the utilization in districts with low to mid-levels of service provision is close to 30%, it increases to roughly 45% in regions with a very high supply density. For men, this rate increases from approximately 24% to about 40% (Figure 4).

## 4. Discussion

In Germany, 11.3% of women and 8.1% of men reported that they had used psychotherapeutic or psychiatric services in the past 12 months. Among women and men with current depressive symptoms, these figures were 35.0% and 31.0%, respectively. In other words, around two thirds of people with current depressive symptoms either chose not to seek professional help during the same 12-month period or were treated by primary carers or somatic care providers. Large regional differences exist in particular in the distribution of outpatient psychotherapeutic care providers. Furthermore, there is a clear correlation between the supply density and the utilization of psychotherapeutic and psychiatric services. In regions with very high psychotherapeutic supply densities, the proportion of people with current depressive symptoms who seek treatment is about 15 percentage points higher than in regions with low coverage. The data demonstrates that not living in marriage/consensual union and having low levels of social support are two factors particularly associated with a higher utilization of services.

The access that people with mental disorders have to the specialised care system differs significantly between regions. The density of psychotherapists varies by a factor of 76.3, and for neurologists/psychiatrists, the density varies by a factor of 17.9, meaning that the density of both groups of professionals far surpasses the rates stipulated as acceptable by the needs-based planning directive (variations by a factor of 2.9 or 2.4) [45]. Furthermore, even if the frequency of mental disorders does not vary dramatically according to region, the consequences of these disorders may still be unequally distributed due to the differences in the density of service provision that exists. ‘Co-provision’ is a term used to describe a situation in which patients in areas with a low supply density visit treatment providers outside of their local area. Once the co-provision of neighbouring regions has been incorporated, the differences between regional supply densities reduce substantially, although they do not completely disappear. Interpretations of co-provision have led to ambiguous findings: on the one hand co-provision mitigates against regional health care disparities and highlights necessary efficient services that centres provide for peripheries. On the other hand, it also makes patient flows visible that were initially triggered by the nominal local shortage in services [46].

Moreover, when interpreting the association between the service supply and the utilization of psychotherapeutic and psychiatric services, it is important to remember that indicators of utilization and regional supply structures can only provide an approximate description of supply provision on the ground. As such, utilization among respondents with current depressive symptoms needs to be treated as a conservative estimate: if current depressive symptoms developed quite recently, the respondent may still not have contacted a psychotherapeutic or psychiatric care provider before the interview. In addition, the groups of physicians included in the GEDA 2014/2015-EHIS questionnaire are not exactly the same as those defined by the needs-based planning directive that are used for the present regional level analyses (see Method). The data from the GEDA 2014/2015-EHIS questionnaire may also include a limited amount of psychological counselling that is provided outside of the medical system. This form of counselling cannot be considered a guideline-compatible form of treatment. At the same time, the densities of neurologists/psychiatrists considered above also include physicians mainly focused on neurological care (as foreseen by the needs-based planning directive for this group of professionals). Importantly, claims data demonstrates that about one third of the most common diagnoses made by neurological care providers actually involve disorders of the nervous system (e.g., Parkinson’s, epilepsy or dementia) [47]. Therefore, this group of physicians is never exclusively available for the treatment of people with mental disorders. As such, a focus on physician densities tends to lead to an overestimation of the medical services that are actually available. Unfortunately, the form in which the data has been collected means that it is impossible to clearly identify which group of physicians a specific care provider belongs to.

Another grey area is the fact that measures of supply density do not adequately reflect the extent of care that is actually being provided. Supply not only depends on the nominal ratio of physicians to the population, but also on various other factors, such as the proportion of physicians who work part-time, the number of patients treated per practice and the frequency of treatment. There are significant differences between the groups of physicians under consideration in this context: claims data from 2008 show that neurological care providers treat a much larger number of cases per quarter than their psychotherapeutic colleagues, but charge for a smaller scope of treatment (time, frequency) per case [47]. As such, both groups of physicians make contributions that are difficult to compare and are very specific in terms of the treatment of mental disorders.

Furthermore, it is important to note that surveys of current depressive symptoms that use the PHQ-8 questionnaire can only provide an approximate indication of psychotherapeutic or psychiatric treatment. It is difficult to directly derive findings about the need for treatment from this data. Current depressive symptoms can occur as an underlying form of depression and may even spontaneously regress; in these cases, there is no need for long-term treatment [48]. However, the target variable does not seek to cover a comprehensive form of therapy, but instead depicts some kind of contact between patients and the health system. In keeping with this, however, the PHQ-8 questionnaire gathers data from a group of people who have an initial need for specialist examination and counselling and, therefore, can certainly be considered in need of care and entitled to services.

Nevertheless, only about one third of people with current depressive symptoms reported having contacted a psychotherapeutic and psychiatric care provider in a 12-month period. This figure corresponds with previous epidemiological findings on utilization that show that a majority of people with clinically relevant depressive disorders go untreated [49]. Claims data from statutory health insurers also demonstrate that when people do seek treatment, about two thirds of individuals with depressive disorders receive no specialist medical examinations and are cared for exclusively by their family physician [5, 11]. Although it is difficult to establish what actually constitutes an ‘appropriate’ level of utilization at the population level, these aspects are certainly relevant in terms of the quality of care. Furthermore, it is likely that depression is under- and over-diagnosed as well as under- and over-treated. One challenge that appears within this context is that between half and two thirds of all diagnoses of depression are unspecific and lack a severity coding; this applies to more than 80% of diagnoses made in general practices [5, 6, 50-53]. Whether these cases can be considered to be guideline-based treatments that would require a precise grading, and frequently specialist assessment, is questionable and should be investigated as part of further studies [11].

When assessing further individual determinants, the differences in utilization according to age and gender are striking. Women usually show higher rates of utilization than men, regardless of the services under consideration [33, 34, 36] and the utilization of psychotherapeutic and psychiatric treatment is no exception [49]. Utilization of psychotherapeutic services tends to decrease with age; this finding is consistent with previous results [34, 54]. In terms of socio-economic status, members of the lower status groups have more frequent contact with psychotherapeutic and psychiatric service providers than those with a medium or high status. However, this difference was not confirmed by the multivariate model once current depressive symptoms had been controlled for. The descriptively higher utilization among people with a low socio-economic status can therefore be attributed to the higher prevalence of current depressive symptoms, and possibly depressive disorders, among this population group [55] (see also the Fact sheets on depression and current depressive symptoms in issue 3/2017 of the Journal of Health Monitoring). Furthermore, having low levels of social support as well as living without a (married) partner, in particular, are independently associated with higher levels of utilization.

This study’s main finding with regard to regional differences is that there is a general correlation between the regional supply of psychotherapeutic care providers (provider density) and the level of utilization among the population. The difference tends to be lower in areas with low to medium supply densities. One possible explanation is that patients in areas with low levels of care provision partially compensate by accepting significantly longer journeys for treatment or switching to inpatient care.

At the same time, it cannot be ruled out that no further increase of utilization is possible once a particular capacity has been reached. As such, it is possible that service providers in regions with lower levels of care provision may treat more patients, but see them less frequently each quarter than in better-served regions. As such, service providers may compensate to a certain extent for the lower supply density. Analyses of claims data suggest that this is happening, and point to regional differences in the number of cases per service provider or in the frequency of treatment in comparisons between eastern and western federal states in Germany [56]. This also ties in with the fact that in the present study the frequency of utilization at the population level hardly differs between the old and new federal states despite considerable differences in the expansion of the outpatient supply structure.

Thus, people with current depressive symptoms living in the roughly 25% of regions with the highest levels of care are currently advantaged in terms of access to care. The significantly higher utilization of psychotherapeutic and psychiatric services in these regions, when it is needed, shows that the people concerned are also making use of this improved access. People living in regions with lower provider densities, on the other hand, remain more often and longer without care or are treated more frequently by specialists in somatic therapy. The extent to which this is reflected in care outcomes, such as more frequent drug therapy, more frequent inpatient admission or increased relapse, is a topic for future research. In any case, these findings – which are consistent with previous studies – militate against the hypothesis of a supplier-induced demand of psychotherapeutic and psychiatric care [19]. In well-supplied regions, utilization increases among people with current depressive symptoms; without these symptoms, it remains at a comparatively low level, despite the better levels of care provision.

One way of achieving a needs-based utilization of psychotherapeutic and psychiatric services involves adapting needs planning and thus improving supply structures. According to the GKV-Versorgungsstärkungsgesetz (GKV-VSG), from 2017 the Federal Joint Committee (G-BA) is to adapt needs planning while taking into account a region’s social and morbidity structure with the particular aim of improving psychotherapeutic care (including at the small area level). Although it became obvious that it can be difficult to find new physicians for practices that need them and that changes in planning do not necessarily lead to improved care, this goal should be pursued further in order to better adapt service provision to the patients’ needs. Doing so improves accessibility by expanding care in areas with poorer levels of provision. Furthermore, it would also reduce the burden faced by the service providers working in these areas and help create more resources for needs-based treatment.

Furthermore, measures need to be considered that can specifically lower the threshold to enabling faster initial contact with health care professionals and to obtaining specialist medical care. The first steps to implementing such changes have already been taken with the amendment of the 2017 psychotherapy directive [57]. Elements such as psychotherapeutic consultations or acute treatment during crises should facilitate earlier access to psychotherapeutic measures in line with the so called stepped care approach. This would also mean that measures could be adapted to reflect individual needs and also help reduce long waiting times which increase the risk of mental disorders becoming chronic. In addition, the discontinuation of elaborate expert assessment procedures for short-term therapies should also enable patients to receive care more rapidly. However, it remains to be seen whether these reforms will merely rearrange existing resources or, ultimately, whether the larger number of subsequent medical examinations will result in more people being classed as in need of treatment.

In accordance with §64b SGB V, various pilot projects are currently testing how the care of people with mental disorders can be improved. In addition to a stronger focus on patients’ needs, the main aim is to strengthen cooperation between primary and specialist care providers in the outpatient and inpatient sectors, and with funding agencies (such as health insurers). These pilot projects often incorporate new forms of care, such as e-mental health, as app and internet-based services can provide low-threshold access to care, help bridge waiting times, as well as improve prevention and promote treatment as part of primary care [58-60]. These measures could be relevant for people in regions with fewer capacities for outpatient treatment and for those who do not use existing services due to a feeling of shame or lack of time. However, these strategies need to be thoroughly tested and evaluated, and are not a substitute for face-to-face psychotherapeutic treatment. Despite this, they demonstrate that improving quality of psychotherapeutic and psychiatric care not only means increasing the number of practitioners (providing greater capacity), but also enhancing and diversifying the ways of access.

## Key statements

- In Germany, the provision of health care for people with mental disorders is an issue of controversial debate.
- Depending on the region in which they live, people with mental disorders have access to very different levels of supply of psychotherapist services.
- 11.3% of women and 8.1% of men report that they have used psychotherapeutic or psychiatric assistance within the last 12 months.
- About two thirds of people with current depressive symptoms have not visited a psychotherapeutic or psychiatric care provider within the last year.
- In regions with relatively good coverage people with current depressive symptoms are much more likely to use the services of psychotherapists.
- Many patients with depressive disorders are exclusively cared for by family practitioners.
- Access to specialist care, as formulated in the stepped care approach, should be further tested alongside increases in the number of care providers.

## Figures and Tables

**Table 1:** Utilization of psychotherapeutic and psychiatric services according to selected social characteristics Source: GEDA 2014/2015-EHIS

	Women		Men	
	%	(95% CI)	%	(95% CI)
**Region**	(n=13,066)		(n=10,809)	
Old federal states (including Berlin)	11.3	(10.4-12.2)	8.0	(7.3-8.7)
New federal states	11.8	(10.4-13.4)	8.7	(7.3-10.3)
**SES**	(n=13,041)		(n=10,782)	
Low	13.7	(11.8-15.8)	10.1	(8.6-11.9)
Middle	10.9	(10.1-11.8)	8.0	(7.1-9,0)
High	9.9	(8.7-11.2)	6.5	(5.5-7.6)
**Health insurance**	(n=12,660)		(n=10,359)	
Statutory	11.3	(10.5-12.1)	8.3	(7.5-9.1)
Private	10.1	(8.6-11.8)	7.0	(5.8-8.4)
**Marriage/consensual union**	(n=12,909)		(n=10,648)	
Yes	9.9	(9.1-10.8)	7.1	(6.5-7.9)
No	13.9	(12.6-15.4)	10.3	(8.9-11.8)
**Social support**	(n=12,851)		(n=10,638)	
Middle/high	9.8	(9.1-10.5)	6.8	(6.2-7.5)
Low	18.1	(16.1-20.4)	13.3	(11.6-15.2)
**Current depressive symptoms**	(n=12,838)		(n=10,653)	
Yes	35.0	(31.8-38.2)	31.0	(27.4-35.0)
No	8.2	(7.5-8.9)	5.9	(5.3-6.6)
**Chronic disease**	(n=12,960)		(n=10,743)	
Yes	16.5	(15.3-17.9)	13.4	(12.3-14.6)
No	6.6	(5.8-7.4)	3.9	(3.3-4.5)

CI=confidence interval; SES=socio-economic status

**Table 2:** Individual and regional determinants for the utilization of psychotherapeutic or psychiatric services; results of logistic multilevel analyses (odds ratios) (n=21,968) Source: GEDA 2014/2015-EHIS

Level	Indicator	Category	OR	95% CI	
Individual	Gender (Ref men)	women	**1.36**	1.21	1.53
	Age (continuous)		**1.0699**	1.0470	1.0933
	Age^2^ (continuous)		**0.9993**	0.9990	0.9995
	SES (Ref low)	middle	1.00	0.84	1.18
		high	0.96	0.76	1.23
	Health insurance (Ref statutory)	private	0.95	0.80	1.12
	Marriage/consensual union (Ref yes)	no	**1.48**	1.30	1.69
	Social support (Ref medium/high)	low support	**1.34**	1.16	1.54
	Current depressive symptoms (Ref no)	yes	**4.85**	4.11	5.72
	Chronic disease (Ref no)	yes	**2.75**	2.38	3.18
District	Psychotherapists per 100,000 IN (continuous)		**1.0071**	1.0014	1.0129
	Neurologists per 100,000 IN (continuous)		0.9855	0.9607	1.0111
	Variation between districts (MOR)		**1.3318**		

SES=socio-economic status; OR=odds ratio; MOR=median odds ratio; Ref=reference category; CI=confidence interval; IN=inhabitants; Psychotherapists, neurologists/psychologists (defined in accordance with the directive for needs-based planning; see Method): corrected for the impact of the co-provision of care

Values in bold: correlation is statistically significant (p<0.05)

## References

[1] Vos T Flaxman AD Naghavi M (2012) Years lived with disability (YLDs) for 1160 sequelae of 289 diseases and injuries 1990-2010: a systematic analysis for the Global Burden of Disease Study 2010. Lancet 380(9859):2163-2196 23245607 10.1016/S0140-6736(12)61729-2 PMC6350784

[2] Institute for Health Metrics and Evaluation (2017) Global Burden of Disease Study 2015 (GBD 2015) Results. Global, both sexes, all ages, YLDs per 100,000. https://vizhub.healthdata.org/gbd-compare/ (As at 20.06.2017)

[3] Institute for Health Metrics and Evaluation (2017) Global Burden of Disease Study 2015 (GBD 2015) Results. Germany, both sexes, all ages, YLDs per 100,000. https://vizhub.healthdata.org/gbd-compare/ (As at 20.06.2017)

[4] Deutsche Angestellten-Krankenkasse (DAK) (2013) DAK-Gesundheitsreport 2013. IGES Institut GmbH, Berlin

[5] Gerste B Roick C (2014) Prävalenz und Inzidenz sowie Versorgung depressiver Erkrankungen in Deutschland - Eine Analyse auf Basis der in Routinedaten dokumentierten Depressionsdiagnosen. In: Klauber J Günster C Gerste B (Eds) Versorgungs-Report 2013/2014. Schattauer, Stuttgart, S. 21-54

[6] Institut für Gesundheits- und Sozialforschung GmbH (IGES) (2012) Bewertung der Kodierqualität von vertragsärztlichen Diagnosen – Eine Studie im Auftrag des GKV-Spitzenverbands in Kooperation mit der BARMER GEK. IGES Institut für Gesundheits- und Sozialforschung GmbH, Berlin

[7] Kliner K Rennert D Richter M (2015) Gesundheit in Regionen – Blickpunkt Psyche. BKK Dachverband, BKK Gesundheitsatlas 2015. MWV Medizinisch Wissenschaftliche Verlagsgesellschaft, Berlin

[8] Techniker Krankenkasse (Ed) (2015) Depressionsatlas. Auswertungen zu Arbeitsunfähigkeit und Arzneiverordnungen. AQUA - Institut für angewandte Qualitätsförderung und Forschung im Gesundheitswesen GmbH, Göttingen

[9] Gesundheitsberichterstattung des Bundes (2017) Rentenzugänge wegen verminderter Erwerbsfähigkeit in der Gesetzlichen Rentenversicherung im Laufe des Berichtsjahres (Anzahl und je 100.000 aktiv Versicherte). Gliederungsmerkmale: Jahre, Region, Zugangsalter, Geschlecht, 1. Diagnose (ICD-10). http://www.gbe-bund.de/ (As at 20.06.2107)

[10] Deutsche Rentenversicherung Bund (2016) Rentenversicherung in Zeitreihen. Ausgabe 2016., DRV-Schriften Band 22.

[11] Deutsche Gesellschaft für Psychiatrie, Psychotherapie und Nervenheilkunde, Bundesärztekammer Arbeitsgemeinschaft der Deutschen Ärztekammern, Kassenärztliche Bundesvereinigung, Arbeitsgemeinschaft der Wissenschaftlichen Medizinischen Fachgesellschaften, Arzneimittelkommission der deutschen Ärzteschaft, Bundespsychotherapeutenkammer Bundesverband der Angehörigen psychisch Kranker, Deutsche Arbeitsgemeinschaft Selbsthilfegruppen, Deutsche Gesellschaft für Allgemeinmedizin und Familienmedizin, Deutsche Gesellschaft für Psychosomatische Medizin und Ärztliche Psychotherapie, Deutsche Gesellschaft für Psychologie, Deutsche Gesellschaft für Rehabilitationswissenschaften (Eds) (2015) S3-Leitlinie/Nationale VersorgungsLeitlinie Unipolare Depression - Langfassung, 1. Auflage. Version 5. 2009, zuletzt verändert: Juni 2015 (AWMF-Register-Nr.: nvl-00). www.depression.versorgungsleitlinien.de (As at 20.06.2017)

[12] Gaebel W Kowitz S Fritze J (2013) Use of health care services by people with mental illness: secondary data from three statutory health insurers and the German Statutory Pension Insurance Scheme. Dtsch Arztebl Int 110(47):799-808 24314623 10.3238/arztebl.2013.0799 PMC3859909

[13] Klose J Rehbein I (2016) Ärzteatlas 2016. Daten zur Versorgungsdichte von Vertragsärzten. Wissenschaftliches Institut der AOK (WIdO), Berlin

[14] Albrecht M Etgeton S Ochmann R (2015) Faktencheck Gesundheit - Regionale Verteilung von Arztsitzen (Ärztedichte). Bertelsmann Stiftung, Gütersloh

[15] Albrecht M Ochmann R Jacobi F (2016) Faktencheck Psychotherapeuten. Faktencheck Gesundheit. Bertelsmann Stiftung und Bundespsychotherapeutenkammer, Berlin

[16] Melchior H Schulz H Härter M (2014) Stellenwert regionaler Variationen in der Prävalenz und Behandlung depressiver Erkrankungen und Implikationen für die Versorgungsforschung. Bundesgesundheitsbl Gesundheitsforsch Gesundheitsschutz 57:224-233 10.1007/s00103-013-1890-3 24469286

[17] Thom J Bretschneider J Müllender S (2015) Regionale Variationen der ambulanten primär- und fachärztlichen Versorgung psychischer Störungen - Regionale Bedarfsunterschiede oder Versorgungsungerechtigkeit? Psychiatrie 12:247-254

[18] Ozegowski S Sundmacher L (2012) Wie „bedarfsgerecht“ ist die Bedarfsplanung? Eine Analyse der regionalen Verteilung der vertragsärztlichen Versorgung. Gesundheitswesen 74:618-626 22886336 10.1055/s-0032-1321748

[19] Jacobi F Becker M Bretschneider J (2016) Ambulante fachärztliche Versorgung psychischer Störungen: Kleine regionale Unterschiede im Bedarf, große regionale Unterschiede in der Versorgungsdichte. Nervenarzt 87(11):1211-1221 27357454 10.1007/s00115-016-0147-4

[20] Jacobi F Barnikol UB (2015) Abschätzung von Prävalenz und Behandlungsbedarf psychischer Störungen: Das Problem diagnostischer Schwellen. Nervenarzt 86(1):42-50 25503066 10.1007/s00115-014-4110-y

[21] Allin S Hernández-Quevedo C Masseria C (2009) Measuring equity of access to health care. In: Smith P Mossialos E Papanicolas I (Eds) Performance measurement for health system improvement Experiences, challenges and prospects. Cambridge University Press, Cambridge, P. 187-221

[22] Bock C Osterkamp N Schulte C (2012) Fachärztliche Versorgung auf dem Land. Mangel oder fehlender Komfort? Gesundheitsmonitor 04/2012. Bertrelsmann Stiftung, Gütersloh

[23] Thode N Bergmann E Kamtsiuris P (2005) Einflussfaktoren auf die ambulante Inanspruchnahme in Deutschland. Bundesgesundheitsbl Gesundheitsforsch Gesundheitsschutz 48(3):296-306 10.1007/s00103-004-1004-3 15768302

[24] Andersen RM (2008) National health surveys and the behavioral model of health services use. Med Care 46(7):647-653 18580382 10.1097/MLR.0b013e31817a835d

[25] Andersen RM (1995) Revisiting the behavioral model and access to medical care: does it matter? J Health Soc Behav 36(1):1-10 7738325

[26] Babitsch B Gohl D von Lengerke T (2012) Re-revisiting Andersen’s Behavioral Model of Health Services Use: a systematic review of studies from 1998-2011. Psychosoc Med 9:Doc11 23133505 10.3205/psm000089 PMC3488807

[27] EuropeanCommission, Eurostat (2013) European Health Interview Survey (EHIS wave 2). Methodological manual. European Commission, Luxembourg

[28] Schulz M Schulz M Bätzing-Feigenbaum J (2015) Vertragsärzte und -psychotherapeuten je 100.000 Einwohner nach Bedarfsplanungsfachgebieten und -regionen im Jahr 2013. Versorgungsatlas-Bericht Nr 15/02. Zentralinstitut für die kassenärztliche Versorgung in Deutschland (Zi), Berlin

[29] Deutsche Rentenversicherung Bund (Ed) (2014) Positionspapier der Deutschen Rentenversicherung zur Bedeutung psychischer Erkrankungen in der Rehabilitation und bei Erwerbsminderung. Deutsche Rentenversicherung Bund, Berlin

[30] Czihal T von Stillfried D Schallock M (2015) Mitversorgungsbeziehungen in der ambulanten Versorgung (Teil 2) - Mitversorgung durch andere Regionen (2008) Versorgungsatlas-Bericht Nr 12/05. Zentralinstitut für die kassenärztliche Versorgung in Deutschland (Zi), Berlin

[31] Czihal T von Stillfried D Schallock M (2015) Mitversorgungsbeziehungen in der ambulanten Versorgung (Teil 1) - Mitversorgung für andere Regionen (2008) Versorgungsatlas-Bericht Nr 12/04. Zentralinstitut für die kassenärztliche Versorgung in Deutschland (Zi), Berlin

[32] Europäische Union (2013) Verordnung (EU) Nr. 141/2013 der Kommission vom 19. Februar 2013 zur Durchführung der Verordnung (EG) Nr. 1338/2008 des Europäischen Parlaments und des Rates zu Gemeinschaftsstatistiken über öffentliche Gesundheit und über Gesundheitsschutz und Sicherheit am Arbeitsplatz in Bezug auf Statistiken auf der Grundlage der Europäischen Gesundheitsumfrage (EHIS). In: Europäische Union (Ed), ABl. L 47 vom 20.2.2013, P. 20-48

[33] Hoebel J Rattay P Prütz F (2016) Socioeconomic Status and Use of Outpatient Medical Care: The Case of Germany. PLoS One 11(5):e0155982 27232878 10.1371/journal.pone.0155982 PMC4883792

[34] Rattay P Butschalowsky H Rommel A (2013) Utilisation of outpatient and inpatient health services in Germany. Results of the German Health Interview and Examination Survey for Adults (DEGS1). Bundesgesundheitsbl Gesundheitsforsch Gesundheitsschutz 56(5-6):832-844 http://edoc.rki.de/oa/articles/reNjmYnmVbkxU/PDF/21x19FTPZ-x3io.pdf (As at 20.06.2017) 10.1007/s00103-013-1665-x 23703505

[35] Kamtsiuris P Bergmann E Rattay P (2007) Inanspruchnahme medizinischer Leistungen. Ergebnisse des Kinder- und Jugendgesundheitssurveys (KiGGS). Bundesgesundheitsbl Gesundheitsforsch Gesundheitsschutz 50(5-6):836-850 10.1007/s00103-007-0247-1 17514470

[36] Rommel A Kroll LE (2017) Individual and Regional Determinants for Physical Therapy Utilization in Germany: Multilevel Analysis of National Survey Data. Phys Ther 97(5):512-523 28340149 10.1093/ptj/pzx022

[37] Lampert T Kroll LE Müters S (2013) Messung des sozioökonomischen Status in der Studie “Gesundheit in Deutschland aktuell” (GEDA). Bundesgesundheitsbl Gesundheitsforsch Gesundheitsschutz 56(1):131-143 10.1007/s00103-012-1583-3 23275948

[38] Dalgard OS Dowrick C Lehtinen V (2006) Negative life events, social support and gender difference in depression: a multinational community survey with data from the ODIN study. Soc Psychiatry Psychiatr Epidemiol 41(6):444-451 16572275 10.1007/s00127-006-0051-5

[39] Kroenke K Strine TW Spitzer RL (2009) The PHQ-8 as a measure of current depression in the general population. J Affect Disord 114(1-3):163-173 18752852 10.1016/j.jad.2008.06.026

[40] Martin A Rief W Klaiberg A (2006) Validity of the Brief Patient Health Questionnaire Mood Scale (PHQ-9) in the general population. Gen Hosp Psychiatry 28(1):71-77 16377369 10.1016/j.genhosppsych.2005.07.003

[41] Lowe B Spitzer RL Grafe K (2004) Comparative validity of three screening questionnaires for DSM-IV depressive disorders and physicians’ diagnoses. J Affect Disord 78(2):131-140 14706723 10.1016/s0165-0327(02)00237-9

[42] Buratta V Frova L Gargiulo L (2003) Development of a common instrument for chronic physical conditions. In: Nosikov A Gudex C (Eds) EUROHIS: developing common instruments for health surveys. IOS press, Amsterdam

[43] Kroll L (2007) XTMRHO: Stata module to calculate intra-class correlations after xtmixed. Statistical Software Components with number S456816. Boston College Department of Economics, Boston

[44] Pfeffermann D Skinner CJ Holmes DJ (1998) Weighting for unequal selection probabilities in multilevel models. J R Stat Soc Series B Stat Methodol 60(1):23-40

[45] Gemeinsamer Bundesausschuss (2016) Richtlinie des Gemeinsamen Bundesausschusses über die Bedarfsplanung sowie die Maßstäbe zur Feststellung von Überversorgung und Unterversorgung in der vertragsärztlichen Versorgung (BedarfsplanungsRichtlinie) in der Neufassung vom 20. Dezember 2012, zuletzt geändert am 15. Dezember 2016. https://www.g-ba.de/downloads/62-492-1408/BPL-RL_2016-12-15_iK-2017-06-01.pdf (As at 20.06.2017)

[46] Czihal T Stillfried D Schallock M (2012) Regionale Mitversorgungsbeziehungen in der ambulanten Versorgung. Zentralinstitut für die kassenärztliche Versorgung in Deutschland (Zi), Berlin

[47] Kruse J Herzog W Hofmann M (2012) Zwischenbericht zum Gutachten “Zur ambulanten psychosomatischen/psychotherapeutischen Versorgung in der kassenärztlichen Versorgung in Deutschland–Formen der Versorgung und ihre Effizienz”. Universitätsklinikum Heidelberg, Universitätsklinikum Gießen und Marburg GmbH, Marburg, Heidelberg

[48] Whiteford HA Harris MG McKeon G (2013) Estimating remission from untreated major depression: a systematic review and meta-analysis. Psychol Med 43(8):1569-1585 22883473 10.1017/S0033291712001717

[49] Mack S Jacobi F Gerschler A (2014) Self-reported utilization of mental health services in the adult German population - evidence for unmet needs? Results of the DEGS1-Mental Health Module (DEGS1-MH). Int J Methods Psychiatr Res 23:289-303 24687693 10.1002/mpr.1438 PMC6878535

[50] Betriebskrankenkassen (BKK) (2015) Gesundheitsatals 2015. Gesundheit in Regionen – Blickpunkt Psyche. BKK Dachverband, BKK Gesundheitsatlas 2015. MWV Medizinisch Wissenschaftliche Verlagsgesellschaft, Berlin

[51] Erhart M Stillfried D (2012) Analyse regionaler Unterschiede in der Prävalenz und Versorgung depressiver Störungen auf Basis vertragsärztlicher Abrechnungsdaten – Teil 1 Prävalenz. Versorgungsatlas. Zentralinstitut für die kassenärztliche Versorgung in Deutschland (Zi), Berlin

[52] Melchior H Schulz H Härter M (2014) Faktencheck Gesundheit - Regionale Unterschiede in der Diagnostik und Behandlung von Depressionen. Bertelsmann Stiftung, Gütersloh

[53] Gaebel W Kowitz S Fritze J (2013) Inanspruchnahme des Versorgungssystems bei psychischen Erkrankungen. Dtsch Arztebl International 110(47):799-808 10.3238/arztebl.2013.0799 PMC3859909 24314623

[54] Schmacke N (2012) Häufigkeit seelischer Erkrankungen. Die Frage nach der “wahren” Prävalenz ist kein akademischer Luxus. GGW 12(3):7-15

[55] Maske UE Buttery AK Beesdo-Baum K (2016) Prevalence and correlates of DSM-IV-TR major depressive disorder, self-reported diagnosed depression and current depressive symptoms among adults in Germany. J Affect Disord 190:167-177 26519637 10.1016/j.jad.2015.10.006

[56] Kassenärztliche Bundesvereinigung (2012) Honorarbericht für das dritte Quartal 2011. Sonderthema: Honorarumsätze im regionalen Vergleich. KBV, Berlin

[57] Gemeinsamer Bundesausschuss (2017) Richtlinie über die Durchführung der Psychotherapie. Fassung vom: 19.02.2009 BAnz. Nr. 58 (P. 1399) vom 17.04.2009. Letzte Änderung: 24.11.2016. In Kraft getreten am: 16.02.2017 https://www.g-ba.de/downloads/62-492-1266/PT-RL_2016-11-24_iK-2017-02-16.pdf (As at 20.06.2017)

[58] Klein JP Gerlinger G Knaevelsrud C (2016) Internetbasierte Interventionen in der Behandlung psychischer Störungen. Nervenarzt 87(11):1185-1193 27649987 10.1007/s00115-016-0217-7

[59] Moessner M Bauer S (2017) E-Mental-Health und internetbasierte Psychotherapie. Psychotherapeut 62(3):251-266

[60] Stein J Rohr S Luck T (2017) Indikationen und Evidenz von international entwickelten Online-Coaches zur Intervention bei psychischen Erkrankungen - ein Meta-Review. Psychiatr Prax 10.1055/s-0043-117050 28851002

[61] Lange C Finger JD Allen J (2017) Implementation of the European health interview survey (EHIS) into the German health update (GEDA). Archives of Public Health 75(1):40 28936356 10.1186/s13690-017-0208-6 PMC5603169

