# Corrigendum: The utilization of psychiatric and psychotherapeutic services in Germany – individual determinants and regional differences

**DOI:** 10.25646/6795

**Published:** 2019-01-31

**Authors:** Alexander Rommel, Julia Bretschneider, Lars Eric Kroll, Franziska Prütz, Julia Thom

Rommel A, Bretschneider J, Kroll LE et al. (2017)

Journal of Health Monitoring 2(4): 3-22.

DOI 10.17886/RKI-GBE-2017-122

An earlier version of this Focus article gave the following incorrect figure on page 11: ‘In fact, the probability that these services will be used increases, on average, by around 7% with the addition of a further ten psychotherapists per district (odds ratio 1.007, Table 2).’ The phrase misstates the average increase in probability of service use (7% instead of the intended 0.7%). The correct sentence reads: ‘In fact, the probability that these services will be used increases, on average, by 0.7% with the addition of a further ten psychotherapists per district (odds ratio 1.007, Table 2).’

The corrected version of the article is available at DOI 10.17886/RKI-GBE-2017-122.2

